# Insulinautoimmunsyndrom: Eine seltene, aber wichtige Differentialdiagnose der Hypoglykämie

**DOI:** 10.1007/s00108-021-01180-0

**Published:** 2021-10-26

**Authors:** Tiago de Castro, Christoph Beier, Christoph Terkamp, Lucia Oehler, Bernhard M. W. Schmidt, Johannes Heck, Dirk Stichtenoth, Heiner Wedemeyer, Holger Leitolf

**Affiliations:** 1 Klinik für Gastroenterologie, Hepatologie und Endokrinologie, Medizinische Hochschule Hannover, Carl-Neuberg-Str. 1, 30625 Hannover, Deutschland; 2 Klinik für Nieren- und Hochdruckerkrankungen, Medizinische Hochschule Hannover, Hannover, Deutschland; 3 Institut für Klinische Pharmakologie, Medizinische Hochschule Hannover, Hannover, Deutschland

**Keywords:** Insulin, Clopidogrel, Hypoglykämie, Hyperinsulinismus, Immunadsorption, Insulin, Clopidogrel, Hypoglycemia, Hyperinsulinism, Immunoadsorption

## Abstract

Eine 69-jährige Patientin mit rezidivierenden schweren Hypoglykämien wurde zur weiteren Diagnostik der Medizinischen Hochschule Hannover zugewiesen. Zuvor hatte die Patientin nach einem Stenting, das aufgrund einer peripheren arteriellen Verschlusskrankheit (pAVK) erforderlich war, mit einer Clopidogreleinnahme begonnen. Das Vorliegen eines Insulinoms und eines paraneoplastischen Syndroms wurde ausgeschlossen. Trotz niedrig normaler Blutzuckerkonzentrationen fanden sich erhöhte Insulin- und Insulinautoantikörperkonzentrationen. Diagnostiziert wurde ein Insulinautoimmunsyndrom, a.e. (am ehesten) ausgelöst durch vorausgegangene Clopidogreleinnahme. Eine Behandlung mithilfe der Immunadsorption wurde initiiert; diese erzielte eine signifikante Reduktion der hypoglykämischen Ereignisse und ein anhaltendes Therapieansprechen über 3 Monate.

## Abstract (en)

A 69-year-old female patient was referred to the Medical University of Hanover for further diagnostic evaluation of recurrent severe hypoglycemia. The patient had previously been started on clopidogrel after arterial stenting for peripheral arterial obstructive disease (PAOD). The presence of an insulinoma and paraneoplastic syndrome was excluded. Increased serum insulin and insulin autoantibodies levels were confirmed, despite normal to low blood sugar levels. An insulin autoimmune syndrome was diagnosed, most likely induced by the prior intake of clopidogrel. Treatment with immunoadsorption was initiated, achieving a significant reduction in hypoglycemic events and a lasting response to treatment over 3 months.

## Anamnese

Eine 69-jährige Patientin wurde als Notfall zur Abklärung rezidivierender symptomatischer Hypoglykämien in der Klinik aufgenommen. Seit 6 Wochen bestünden autonome und auch neuroglykopene Symptome einer Hypoglykämie wie Zittern, Kaltschweißigkeit, Präsynkopen und Desorientiertheit. In der Häuslichkeit wurde ein fremd beobachteter Krampfanfall dokumentiert, die daraufhin gemessene Blutzuckerkonzentration betrug 2,28 mmol/l. Ein Diabetes mellitus war nicht bekannt, und eine exogene Insulinapplikation wurde ausgeschlossen. Die Patientin erhielt im Verlauf ein Messsystem zu kontinuierlicher Glucosemessung, eigenständiger Detektion drohender Hypoglykämien und Erhebung eines glykämischen Tagesprofils. Als Vorerkrankungen waren eine essenzielle Hypertonie, eine mittelschwere chronisch obstruktive Lungenerkrankung (COPD) mit Emphysem und fortgesetztem Nikotinabusus sowie eine periphere arterielle Verschlusskrankheit (pAVK) mit Zustand nach Stent-Implantation bekannt. Aufgrund der erst kürzlich erfolgten Stent-Implantation zur Behandlung der pAVK nahm die Patientin seit 10 Wochen eine duale Thrombozytenaggregationshemmung mit Acetylsalicylsäure 100 mg/Tag und Clopidogrel 75 mg/Tag ein. Darüber hinaus lag eine Adipositas Grad I (Body-Mass-Index [BMI] 32,5 kg/m^2^) vor.

## Untersuchungsbefunde

Aufgrund der präsynkopalen Zustände und eines Krampfanfalls waren bereits zweimalige notfallmäßige Einweisungen in externe Krankenhäuser erfolgt, in denen eine kardiologische und neurologische Ursache der oben genannten Symptomatik ausgeschlossen werden konnte. Das Vorliegen einer Whipple-Trias konnte klinisch wiederholt bestätigt werden. Simultan zum klinischen und zum laborchemischen Nachweis von Hypoglykämien (Blutglucosekonzentration 3,33 mmol/l ± 1,41 mmol/l) konnten wiederholt sowohl deutlich erhöhte Insulinspiegel (> 1000 mU/l, Normbereich 2,6–24,9 mU/l) sowie Insulinautoantikörper (> 100 IU/ml, Normbereich bis 10 IU/ml) bei inadäquat niedriger C‑Peptid-Konzentration (2,27 nmol/l ± 0,63 nmol/l, Normbereich 1,1–4,4 ng/ml) nachgewiesen werden. Der HbA_1c_-Wert betrug 5,7 % (Normbereich 4,8–5,6 %) und lag somit formal im prädiabetischem Bereich. Biochemisch ergaben sich keine wegweisenden Hinweise auf klassische endokrinologische Ursachen der Hypoglykämien. Während eines Fastentests kam es erst nach 72 h zu einer Hypoglykämie. Eine tumorsuspekte Raumforderung ließ sich weder in der konventionellen Schnittbildgebung noch in einer funktionellen Bildgebung mittels Gallium 68-DOTA-TATE Positronenemissionstomographie mit CT-Koregistrierung (*Ga68-*DOTA-TATE PET/CT) nachweisen.

## Diagnose

- Aufgrund des klinischen und laborchemischen Verdachts auf ein a.e. medikamentös induziertes Insulinautoimmunsyndrom (IAS) wurde die Medikationsliste der Patientin an das Institut für Klinische Pharmakologie übersendet; dort konnte Clopidogrel als mögliche auslösende Substanz benannt werden. Besonders hinweisend war der zeitliche Verlauf mit Beginn der hypoglykämischen Symptomatik ca. 4 Wochen nach der Therapieeinleitung von Clopidogrel. Clopidogrel ist ein Pro-Drug, das nach enzymatischer Aktivierung eine Sulfhydrylgruppe als aktiven Metaboliten enthält. Sulfhydrylgruppen stehen im Verdacht, mit der Disulfidbindung des Insulins zu interagieren, wodurch Insulin immunogener wird und folglich vermehrt Insulinantikörper produziert werden [1].

## Therapie und Verlauf

**Figure 1:**
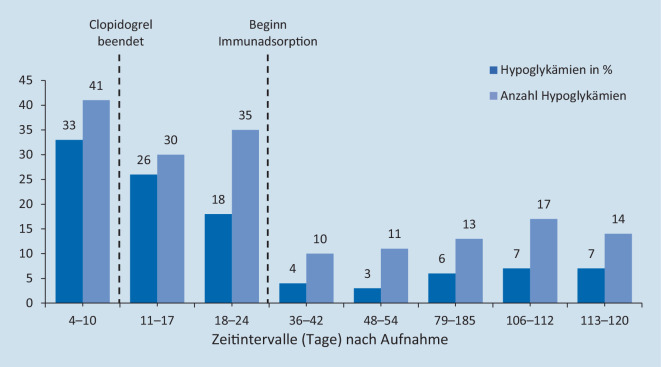

**Figure 2:**
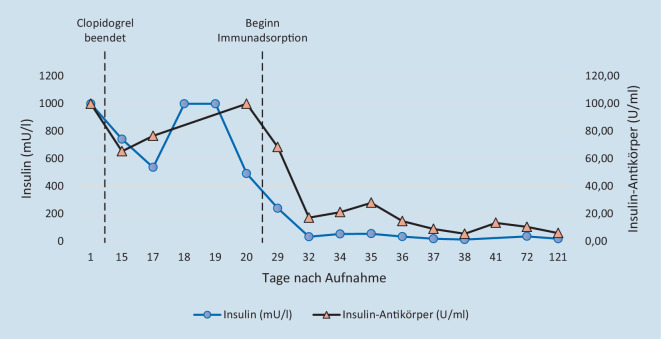

Trotz Beendigung der Therapie mit Clopidogrel und Einleitung unterstützender diätetischer Maßnahmen sistierten die Hypoglykämien nicht (Abb. 1), sodass eine Behandlung mithilfe der Immunadsorption eingeleitet wurde. Eingesetzt wurde bei der Patientin ein regenerierbarer GLOBAFFIN®-Filter mit Peptid-GAM®-Liganden (Fresenius Medical Care, Bad Homburg). Nach 6‑maliger Immunadsorption konnte eine deutliche Reduktion der Hypoglykämien erreicht werden (Abb. 1). Zeitgleich normalisierten sich sowohl der Insulin- als auch der Insulinautoantikörperspiegel (Abb. 2). Somit war eine Entlassung mit fortlaufender eigenständiger Glucosesensormessung möglich. Anschließende ambulante Verlaufskontrollen über einen Zeitraum von 3 Monaten ergaben keinen erneuten Hinweis auf symptomatische Hypoglykämien (Abb. 1).

## Diskussion

Das IAS ist eine seltene Erkrankung, charakterisiert durch hyperinsulinämische Hypoglykämie und erhöhte Insulinautoantikörper(IAA)-Titer bei Ausschluss einer exogenen Insulinapplikation, eines Insulinoms oder extrapankreatischer Neoplasien [2]. Erstmals beschrieben wurde die Erkrankung 1970 durch Hirata et al. [3]. Internationale epidemiologische Kennzahlen sind aufgrund der geringen Inzidenz unklar; über 90 % der Fälle wurden in Japan beschrieben. Die Erkrankung trifft beide Geschlechter mit etwa gleicher Häufigkeit und betrifft v. a. Patienten über dem 40. Lebensjahr [4]. Eine ungleiche geografische Inzidenz des IAS wird u. a. auf eine „Human-leukocyte-antigen“(HLA)-DR4-Assoziation zurückgeführt [5].

Häufig wird eine Assoziation mit Medikamenten oder Nahrungsergänzungsmitteln mit Sulfhydrylgruppen (Tab. 1), viralen Infektionen und Autoimmunerkrankungen berichtet [6, 7]. Pathophysiologisch wird ein Zwei-Phasen-Mechanismus diskutiert, in dem Insulin mit Insulinautoantikörpern eine Immunkomplexbindung eingeht und somit eine Bindung von Insulin an den Insulinrezeptor verhindert wird [2]. Initial kommt es hierdurch zu milden Hyperglykämien, gefolgt von kompensatorischer Insulinfreisetzung aus den β‑Zellen mit zeitgleicher Insulinfreisetzung aus den gebildeten Immunkomplexen und resultierenden Hypoglykämien.

**Table 1:** 

Wirkstoffklasse	Wirkstoff	Stärke der Evidenz
Thyreostatika	Thiamazol	Hoch
	Carbimazol	Mittel
Nahrungsergänzungsmittel	α‑Liponsäure	Hoch
	Pyritinol	Niedrig
	Glutathion	Niedrig
	Methionin	Niedrig
Antihypertensiva und Antiarrhythmika	Captopril	Niedrig
	Hydralazin	Niedrig
	Procainamid	Niedrig
	Diltiazem	Niedrig
Thrombozytenaggregationshemmer	Clopidogrel	Niedrig
Orale Antidiabetika	Tolbutamid	Niedrig
	Gliclazid	Niedrig
Antiphlogistika	Steroide	Niedrig
	Loxoprofen	Niedrig
	Diclofenac	Niedrig
Muskelrelaxanzien	Tolperison	Niedrig
Antibiotika	Imipenem	Niedrig
	Penicillin G	Niedrig
	Isoniazid	Niedrig
Protonenpumpenhemmer	Pantoprazol	Niedrig
	Omeprazol	Niedrig
Plasmaproteine	Albumin	Niedrig
Chelatbildner	Tiopronin	Niedrig
	Penicillamin	Niedrig

Aufgrund der gestörten Insulinrezeptoraktivierung kommt es zur kompensatorischen Insulinfreisetzung

Diätetische Maßnahmen mit häufigen kleinen, kohlenhydratarmen Mahlzeiten können therapeutisch bei oft selbstlimitierendem Verlauf nach Expositionsbeendigung der auslösenden Noxe ausreichen. In Fallberichten wurden pharmakologische (Acarbose, Somatostatinanaloga, Diazoxid) und immunsuppressive Therapieansätze, vorwiegend Glukokortikoide und Azathioprin [4], dargestellt. Ebenfalls ist der erfolgreiche Einsatz von Rituximab zur selektiven Depletion CD20-positiver B‑Zellen bei IAS in der Literatur beschrieben [8, 9]. Bei schwersten Verläufen wurden auch plasmapheretische Verfahren eingesetzt [10]. In einem Fallbericht wurden bei glukokortikoid- und azathioprinrefraktärem IAS 10 Immunadsorptionen, gefolgt von einer Erhaltungstherapie mit Rituximab, erfolgreich angewendet [9].

Bei der vorgestellten Patientin konnte die auslösende Medikation umgehend abgesetzt und dadurch eine prolongierte Exposition verhindert werden. Aufgrund des ausbleibenden Erfolgs diätetischer Maßnahmen und des erwartbar ungünstigen Nutzen-Risiko-Profils einer immunsupprimierenden bzw. immunmodulierenden Therapie, wurde die Durchführung einer Immunadsorption bevorzugt, wodurch eine rasche Reduktion symptomatischer Hypoglykämien erreicht werden konnte. In weiteren ambulanten Verlaufskontrollen war das Therapieansprechen über 3 Monate anhaltend.

## Fazit für die Praxis

- Das Insulinautoimmunsyndrom (IAS) stellt eine seltene, vermutlich unterdiagnostizierte, aber dennoch klinisch relevante Differenzialdiagnose der Hypoglykämie dar. Differenzialdiagnostisch wegweisend ist die Konstellation aus Hypoglykämie und Hyperinsulinämie sowie dem Nachweis niedrig-normaler C-Peptid-Spiegel sowie erhöhter Insulin-Autoantikörper.
- Eine rechtzeitige Diagnosestellung kann unnötige und kostspielige Diagnostik sowie nichtindizierte Therapien bis hin zu einer Pankreasresektion verhindern.
- Nach bestätigter Diagnose eines IAS ist in Ermangelung einer etablierten Standardtherapie die Indikationsstellung für eine bestimmte Therapieform anhand einer strengen Nutzen-Risiko-Bewertung patientenindividuell zu treffen. Trotz zunehmender wissenschaftlicher Erkenntnisse über das IAS fehlen aktuell noch weitestgehend Informationen zu den verschiedenen Behandlungsansätzen. Weiterführende kontrollierte klinische Studien sind dringend erforderlich, wobei die geringe Inzidenz des IAS eine Rekrutierung von Studienteilnehmern erheblich erschweren dürfte.
